# HLA-DQ2 and -DQ8 genotype frequency in Syrian celiac disease children: HLA-DQ relative risks evaluation

**DOI:** 10.1186/s12876-018-0802-2

**Published:** 2018-05-24

**Authors:** Hossam Murad, Batoul Jazairi, Issam Khansaa, Doaa Olabi, Lina Khouri

**Affiliations:** 1 Molecular Biology and Biotechnology Department, Human Genetics Division, Atomic Energy Commission of Syria, P.O. Box 6091, Damascus, Syria; 2 Children’s hospital, Damascus, Syria

**Keywords:** Celiac disease, HLA, Children, Syria

## Abstract

**Background:**

Celiac disease (CD) is a common autoimmune disease in Syria which manifesting with inflammation of the small intestine and with various extra intestinal symptoms. The disease is associated with human HLA-DQ genes encoding HLA-DQ2 and DQ8 proteins.

**Methods:**

In this study, 49 children patients of CD and 58 healthy control samples were genotyped for HLA-DQ genes using SSP-PCR technique. Relative risks for different genotypes were also evaluated.

**Results:**

The DQB1*0201 allele was the most common in the patients (77.6%) followed by DQB1*0302 allele (10.2%). The highest HLA-DQB risk for CD development was found in patients carriers a DQ2.5/DQ8 genotype (1/10), followed by the patients carriers DQ2.5/DQ2.5 (1/12).

**Conclusion:**

The significant differences in the frequency of HLA-DQ2 and HLA-DQ8 in Syrian patients in compared with controls and relative risks predicted demonstrated the importance role of these alleles in the development of CD in Syrian children patients.

## Background

Celiac disease is an immune-mediated disorder with a strong genetic predisposition and dietary gluten as an environmental trigger [1]. This disease causes inflammation in the small intestinal mucosa. The developing villus atrophy can impede the absorption of nutrients [2].

Several ways to diagnosis celiac disease using anti-tissue transglutaminase (anti-TG2) test, anti-endomysium (EMA) auto-antibodies and small bowel biopsy [3]. This disease is widespread in most Mediterranean countries [4], and it estimated that about more than 5 million patients will be affected in the next 10 years in the Mediterranean region [5]. The only effective therapy is gluten-free diet for life-long, which leads to complete remission of all clinical signs [6, 7]. Celia disease characterized by an interaction of a certain genes, gluten, and environmental influences [8]. The majority of patients with CD carrying the HLA class II genes: HLA-DQ2.5 (DQA1*05-DQB1*02) and HLA-DQ8 (DQA1*03-DQB1*0302) [9]. Strong association was observed between HLA-DQ2.5 and predisposition to CD, explained by its affinity to binding gluten proteins [10]. While, HLA-DQ2.2 contributes less to the risk of CD [11]. Moreover, the risk of CD is shown to be higher in individuals homozygous for the HLA-DQ2.5 or HLA-DQ2.5/DQ2.2 genotypes compared with those homozygous for HLA-DQ2.2 or heterozygous for HLA-DQ2.5 or DQ2.2 [12, 13].

HLA genotyping can be used as an efficient adjunct in the CD diagnostic as already confirmed by the New ESPGHAN guidelines for the diagnosis of CD [14, 15].

In Syria, previous reports indicated that Syrian population has high prevalence of the CD (1/62) in healthy blood donors [16, 17], but no published study about the link of HLA-DQ alleles and Syrian celiac patients; so, we aim to study the distribution of the CD genes: DQ2 and DQ8 in 49 children patients (age from 1 to 18 years old) in comparable with 58 control samples of the same ages.

## Methods

### Patients and controls

Forty nine CD patients (35 males and 14 females; mean age, 9.5 years; range (1–18) and fifty eight healthy controls (30 males and 28 females; mean age, 29 years; range, (18–40) with no history of CD, cancer or autoimmune diseases and were negative for CD serological screening, were selected from the blood transfusion center of Damascus University in Syria. All patients had positive tTGA and/or EMA antibodies and histology according to Rostami Marsh classification [3, 18].

This study was approved by the Ethics Committee of Atomic Energy Commission of Syria, and written informed consent was obtained from all participants. If participant was a minor, parental consent was taken.

### HLA DQ2 and DQ8 genotyping

Peripheral blood was drawn on BD Vacutainer plus Blood Collection tubes K2 EDTA 18.0 mg (BD Biosciences, USA). Genomic DNA was extracted from peripheral blood cells using the salting-out method [19]. HLA-DQB1 genotype was performed by sequence-specific primer-polymerase chain reaction (SSP-PCR) method using published primers [20] and for HLA-DQA1 genotype [21].

### Statistical analysis

Data were analyzed using SPSS software (version 22, SPSS Inc., Chicago, Illinois, USA). Odds ratios was applied to establish statistical significance with 95% confidence (*P* value ≤0.05). Relative risk of developing disease for specific genotypes were calculated as (% of allele in patients / % of allele in controls) x probability to be affected in general population [22].

## Results

In this study, forty nine definite CD patients (25 males and 24 females) and 58 controls of Syrian origin were genotyped for HLA-DQ2 and DQ8 genes. The allele and genotype frequencies of DQ2 and DQ8 genes in CD were compared with the controls. The frequency of the DQB1*02:01 allele was very significantly increased in patients compared with the controls (77.6% vs. 58.6%; *p* value: 0.00). The frequency of the DQB1*03:02 allele was also elevated in patients versus controls, but uncorrected *P* value was borderline significant (10.2% vs. 8.6%; *p* value: 0.09), Table 1).

**Table 1:** HLA-DQ2 (DQB1*0201) and HLA-DQ8 (DQB1*0302) alleles frequency in patients and control

Allele	Patient (*n* = 49) (98 alleles)		Control (*n* = 58) (116 alleles)		*P*-value
	*n*	Fr (%)	*n*	Fr (%)	
DQB1*0201	76	77.6	34	29.3	< 0.01
DQB1*0302	10	10.2	5	4.3	0.09
DQ7	8	8.2	4	3.4	0.13
DQX	4	4.1	73	62.9	< 0.01

*Fr* frequency. *P*-values are results of Chi-square test; significance level is defined at ≤ 0.05

Genotype analysis of DQ2 and DQ8 genes shows that the 24 of 49 patients carrying DQ2.5 in homozygous genotype with high frequency 49%, whereas, this genotype was found in 6 controls (10.3%). The frequency of the DQ2.5/DQ2.2 or DQ2.5/DQ8 were increased in patients versus controls (10.2% vs. 3.4%), (10.2% vs. 1.7%) respectively. More genotype analysis demonstrated that 30.6% of patients carried another DQ genotypes, while 84.5% of controls had these genotypes (Table 2).

**Table 2:** Distribution of HLA-DQ genotypes in patients with celiac disease and control

HLA Genotype	Patient (*n* = 49)		Control (*n* = 58)		Odds ratio	95% CL	Relative risk	*P*-value
	*n*	Fr (%)	*n*	Fr (%)				
DQ2.5/DQ8	5	10.2	1	1.7	6.48	0.73–57.46	1/10	0.09
DQ2.5/DQ2.5	24	49	6	10.3	8.32	3.02–22.93	1/12.5	< 0.01
DQ2.5/DQ2.2	5	10.2	2	3.4	3.18	0.59–17.19	1/20	0.18
DQ8/DQX	4	8.2	2	3.4	2.49	0.44–14.21	1/25	0.31
DQ2.5/DQ7	8	16.3	4	6.9	2.63	0.74–9.35	1/25	0.13
DQ2.2/DQ8	1	2	1	1.7	1.19	0.07–19.92	1/50	0.90
DQ2.2/DQ2.2	2	4.1	3	5.2	0.78	0.13–4.87	1/100	0.79
DQ2.x	0	0	4	6.9	0.12	0.01–2.33	0	0.16
DQX/DQX	0	0	35	60.3	0.01	0.00–0.11	0	< 0.01

*n* number of the patients or the control, *Fr* frequency. Odds ratio and *P*-values have been calculated on the total data set comparing CD patients versus the control genotypes. Relative risk of developing disease for specific genotypes were calculated as a ratio of specific HLA-DQ genotype proportion in CD patients and control by taking into account the prevalence of the CD (0.016) in Syrian population

The highest HLA-DQB relative risk for CD development was found in patients carriers DQ2.5/DQ8 genotype (1/10), while, the patients carriers of DQ2.5/DQ2.5 or DQ2.5/DQ2.2 genotype had a relative risk about 1/12.5 and 1/20 respectively (Table 2).

Based on the Marsh Score, forty-two of 49 CD patients with biopsy result were classified into Marsh I, Marsh II and Marsh III as indicated in Table 3.

**Table 3:** Distribution of HLA-DQ2 and DQ8 alleles according to severity of mucosal damage

Marsh classification	Patient (*n* = 42)				*P*-value
	DQ2		DQ8		
	*n*	Fr (%)	*n*	Fr (%)	
Marsh I	4	9.5	0	0	0.04
Marsh II	10	23.8	2	4.8	0.01
Marsh III	22	52.4	4	9.5	< 0.01

*n* number of the patients, *Fr* frequency

The frequency of the patients with mild form of CD (Marsh I) carrying DQ2 allele was 9.5%, whereas, the frequency was 23.8% for the patients with Marsh II having DQ2. While, 26 patients from 42 (52.4%) were classified into Marsh III, 22 out of them carrying DQ2 allele, all this results indicated the involvement of DQ2 allele in the severity of mucosal damage (Table 3).

## Discussion

Celiac disease is a systemic immune mediated disorder caused by the intolerance of gluten containing grains in genetically susceptible person [23]. It was found that there is a significant relation between the wheat consumption and the frequency of HLA alleles DQ2 and DQ8 worldwide [24]. The history of CD is backed to the spreading of wheat cultivation after the agricultural revolution.

The pathogenesis of CD is dependent on the presence of HLA class II genes: HLA- DQ2 (DQA1*05-DQB1*02) and HLA-DQ8 (HLA-DQA1*0301-DQB1*0302) which are specific to gluten [25]. In contrast, the absence of either HLA-DQ2 or HLA-DQ8 has a negative predictive value of nearly 100% in excluding the diagnosis of celiac [8].

This study demonstrated that, among 49 Syrian children patients from the Children’s Hospital, Damascus, Syria there were 77.6% carried the DQB1*0201 allele and 10.2% carrying the DQB1*0302 allele. All patients studied that carried two DQ2 or DQ8 alleles had a common severe symptoms (fatigue, weight loss, stunted growth, iron deficiency anemia and chronic abdominal pain) and they had high value of anti-transglutaminase antibody, and most of them had classified according to the March classification and villous atrophy of variable severity [26].

Our results indicated a presence of high significant association between DQB1*02;01 allele and CD development, while, it was a borderline significant association with DQB1*03:02 allele. The results also shown that 87.8% of CD patients carried at least one of DQB1*02:01 or DQB1*03:02 alleles. All these results indicated that there were a high statistically significant association between these two alleles and the Syrians CD patients. This data is in concordance with the results obtained for CD patients from several Arab countries regarding HLA-DQ2 variants: in Egyptian (77.42%); Gaza strip patients (84.6%); Jordanian (100%) and Moroccan CD patients (45.2%) [27–30]. In the other hand, this HLA-DQ2 variants had also high frequented in French (87%), Italian (84%), and in UK (88%) CD patients [31].

We have evaluated the relative risk for different HLA genotypes (Table 2). The highest HLA-DQ genotype relative risk for CD in our patients was associated with DQ2.5/DQ8 genotype carriers (1/10). Also, carriers of DQ2.5/DQ2.5 genotype were in group with high relative risk (1/12.5). Recent published studies also confirmed that DQ2.5/DQ2.5 genotype conferred the highest risk for CD [32–34]. In the other hands, in our group, the patients carried DQ2.5/DQ2.2 or DQ8/DQX or DQ2.5/DQ7 genotypes showed medium risks for CD (1/20 and 1/25) respectively. The lowest risk was detected in patients of DQ2.2/DQ8 or DQ2.2/DQ2.2 genotype carriers. So, patients with a one dose or double dose of HLA-DQ2.5 have high risk for CD, this data is largely consistent with published data about the dose effect for DQB1*02 allele for development of CD [12].

In our study, the frequency of CD patients with Marsh I and Marsh II carried DQ2 allele was 9.5 and 23.8% respectively, while, it reached 4.8% in the CD patients carried DQ8. In the other hand, Marsh III score was given more than fivefold higher in the CD patients carried DQ2 (52.4%) in compare with CD patients carried DQ8 (9.5%). These results presenting high positive association of DQ2 allele with Marsh III of CD patients. Several previous studies have demonstrated the role of the DQ2 allele in the severity of mucosal damage [35, 36], where, Zamani et al., revealed that 02:01 allele had a significant association with Marsh IIIc patients (pc: 0.02, OR: 14.55) and it represented involvement of this allele in the severity of mucosal damage [34].

## Conclusion

We studied for the first time the distribution of HLA-DQ genotypes in the children celiac patients and we estimated the risk for CD development. The significant differences in the frequency of HLA-DQ2 and HLA-DQ8 alleles in Syrian patients compared with controls demonstrated the importance role of these alleles in the development of CD and support the possibility of using HLA-DQ typing in confirmation of the disease.

## Abbreviations

Anti-TG2: Anti-tissue transglutaminase
CD: Celiac disease
EMA: Endomysial antibody
HLA: Human leukocyte antigen
SSP-PCR: Single Specific Primer-Polymerase Chain Reaction
